# Cost-effectiveness analysis of NEPA, a fixed-dose combination of netupitant and palonosetron, for the prevention of highly emetogenic chemotherapy-induced nausea and vomiting: an international perspective

**DOI:** 10.1007/s00520-022-07339-1

**Published:** 2022-09-08

**Authors:** Jonas Nilsson, Vittoria Piovesana, Marco Turini, Claudio Lezzi, Jennifer Eriksson, Matti Aapro

**Affiliations:** 1 ICON Plc, Stockholm, Sweden; 2 Helsinn Healthcare SA, Lugano, Switzerland; 3 Genolier Cancer Centre, Clinique de Genolier, Genolier, Switzerland

**Keywords:** Chemotherapy-induced nausea and vomiting, Cost-effectiveness, Antiemetics, Netupitant, Palonosetron, NEPA

## Abstract

**Purpose:**

The aim of this study was to assess the cost-effectiveness of NEPA, a fixed-dose combination of oral netupitant (300 mg) and palonosetron (0.5 mg), compared to available treatments in Spain after aprepitant generic introduction in the market, and to discuss results in previously performed analyses in different wordwide settings.

**Methods:**

A Markov model including three health states, complete protection, complete response at best and incomplete response, was used to evaluate the cost-effectiveness of NEPA versus common treatment options in Spain during 5 days after chemotherapy. Incremental costs including treatment costs and treatment failure management cost as well as incremental effects including quality adjusted life days (QALDs) and emesis-free days were compared between NEPA and the comparator arms. The primary outcomes were cost per avoided emetic event and cost per QALDs gained.

**Results:**

NEPA was dominant (more effective and less costly) against aprepitant combined with palonosetron, and fosaprepitant combined with granisetron, while, compared to generic aprepitant plus ondansetron, NEPA showed an incremental cost per avoided emetic event of €33 and cost per QALD gained of €125.

**Conclusion:**

By most evaluations, NEPA is a dominant or cost-effective treatment alternative to current antiemetic standards of care in Spain during the first 5 days of chemotherapy treatment in cancer patients, despite the introduction of generics. These results are in line with previously reported analyses throughout different international settings.

## Introduction

Chemotherapy-induced nausea and vomiting (CINV) is ranked by patients as one of the most distressing side effects cancer patients experience during chemotherapy [30, 42] and can negatively impact quality of life and the ability to carry out the activities of daily living [18]. Based on the Functional Living Index Emesis (FLIE) questionnaire, 37.2% of all patients reported reduced daily functioning, and of those with poorly managed CINV, about 90% reported a significant impact on daily functioning [22].

A survey among women with breast cancer showed that patients would, on average, risk a 38% chance of being dead to avoid having grade III/IV nausea/vomiting for the rest of their lives, signalling the importance of effective prophylactic treatments for these patients [30]. Experiencing CINV side effects is not only debilitating to patients but is frequently cited as a major reason for treatment discontinuation [43].

CINV is classified according to time of onset after chemotherapy administration into acute (occurring within the first 24 h), delayed (between 24 and 120 h) and overall (between 0 and 120 h) phase and may last for several days [7, 27]. Without prophylactic treatment, it is estimated that over 90% of patients exposed to highly emetogenic chemotherapy (HEC) and between 30 and 90% of patients exposed to moderately emetogenic chemotherapy (MEC) will experience acute-phase CINV [38]. Cancer drugs are classified as either low, minimal, moderate or highly emetogenic risk, based on the risk of vomiting without any antiemetic prophylaxis. Low risk is assigned to 10–30%, moderate risk to 30–90% and high risk > 90% incidence of vomiting [23]. CINV is associated with certain chemotherapy agents, e.g., HEC drugs such as cyclophosphamide (> 1500 mg/m^2^), cisplatin and carmustine and MEC drugs including doxorubicin, cyclophosphamide (< 1500 mg/m^2^), epirubicin and oxaliplatin.

While the emetogenic potential of chemotherapy agents is predictive of CINV [23], several patient-related risk factors have also been identified [26], including occurrence of CINV in previous cycle and its duration [34, 40].

Despite the introduction of more effective antiemetics, up to 20% of cancer patients treated with HEC still suffer from moderate to severe CINV (≥ grade 2) [14]. Other analysis showed, for patients receiving MEC, that despite the use of antiemetic prophylaxis, 20.8% of patients experienced at least one episode of vomiting, 42% nausea of any intensity and significant nausea in 23.8% of the patients [16].

Poor adherence to recommended prophylaxis has been reported, in several observational studies [4–6, 17]. Suboptimal adherence to prophylaxis may lead to uncontrolled CINV [28] with significant impact not only on patient’s quality of life but also on CINV-related direct costs such as acquisition cost of antiemetic drugs and rescue medication, administration devices, add-on treatments, nursing and physician time, unscheduled office visits, emergency room admissions and, in some cases, extended hospitalization or readmission [25, 35, 41]; this leads to an increased economic burden to healthcare systems, as shown in various international studies.

A study conducted in the USA describing why patients with cancer use the emergency department (ED) identified nausea and vomiting as one of the main reasons for ED visits. Of 37,760 visits, 2543 were attributable to nausea and vomiting [33].

One retrospective study analysed costs associated with CINV in patients with cancer, treated with HEC, MEC, or LEC in the outpatient setting [15]. The cost of inpatient, outpatient and ER visits and pharmacy costs (rescue medications for CINV treatment) were included. Despite prophylactic treatment, in the follow-up period, a total of 47,988 CINV events occurred with an associated all-cause treatment cost of US $89 million. The average daily CINV treatment cost for all care settings was US $1855.

A retrospective claims analysis found that patients treated with chemotherapy that experienced CINV had significantly higher CINV-related costs compared to those without CINV ($2058 and $139, respectively). Furthermore, those patients with CINV-related claims had significantly higher total direct healthcare costs compared to those with no CINV-related claims [29].

Burke et al. analysed 19,139 patients treated at 257 outpatient hospital facilities in the USA who had received HEC (16%) or MEC (84%) [12]. All patients received at least one antiemetic agent at the chemotherapy administration visit (the most common antiemetic therapies listed were 5-HT_3_ antagonists and corticosteroids). Approximately one in eight patients had a follow-up hospital visit associated with CINV after a first cycle of HEC or MEC. A total of 2641 patients (13.8%) experienced one or more CINV-associated hospital visit after a first cycle of HEC or MEC. Inpatient admissions (64%) were the most common type of hospital visit and were also the most costly type of visit, averaging approximately US$7500 per patient.

From a healthcare payer’s perspective, there is a need to ensure adherence to guidelines [38].

Multinational Association of Supportive Care in Cancer/European Society for Medical Oncology (MASCC/ESMO) antiemetic guidelines recommend a triplet prophylaxis with a neurokinin-1 receptor antagonist (NK_1_ RA), a 5-hydroxytryptamine-3 receptor antagonist (5-HT_3_ RA) and dexamethasone for patients receiving HEC, including anthracycline-cyclophosphamide (AC) and carboplatin (considered MEC) based chemotherapy [1, 7]. Olanzapine may be added to the triplet when the occurence of nausea associated with HEC and AC regimens is an issue [7].

Adding more liberally an NK_1_RA according to international guidelines’ recommendations suggests a potential reduction of healthcare resource consumption due to uncontrolled CINV.

The aim of this study was to assess the cost-effectiveness of NEPA, compared to available treatments in Spain for patients receiving HEC and to discuss results in previously performed analyses in different wordwide settings.

## Methods

**Fig. 1:**
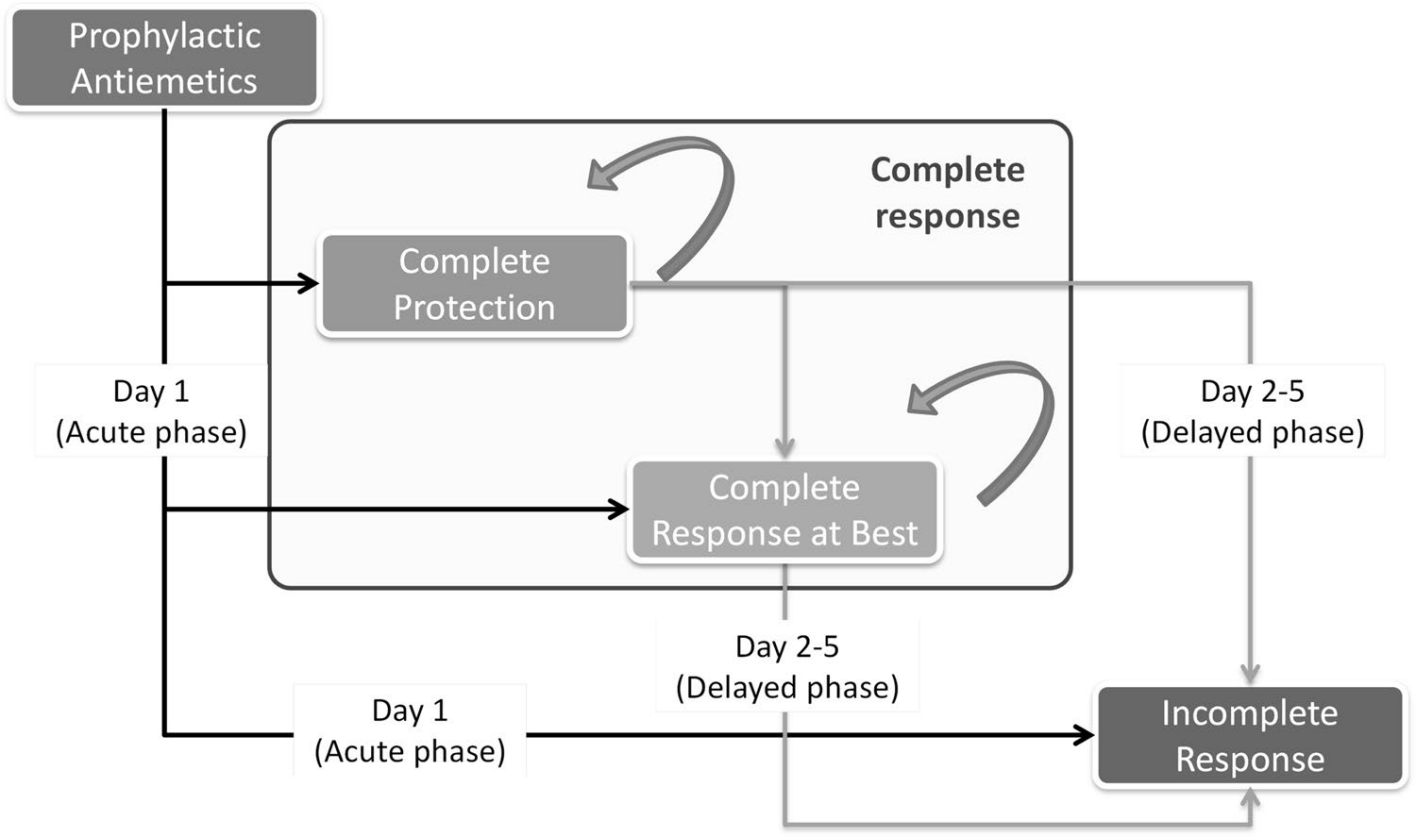
Markov model illustrating the three health states: complete protection (CP), complete response at best (CR) and incomplete response (no CR). Note: Complete protection indicates less than 25 mm on VAS (no significant/mild nausea) without emesis and rescue medication. Complete response at best indicates at 25 mm or more on VAS without emesis and rescue medication. Incomplete response indicates experience of emesis episodes and/or rescue medications

The targeted patient population for the analysis was cancer patients receiving prophylactic antiemetics for the management of HEC. A Markov model, previously developed for the UK [13], was adapted for Spain [15]. A 5‐day time horizon was adopted, consisting of the first day (acute phase) and a delayed phase (days 2–5), and was run for one cycle of chemotherapy (Fig. 1) [15]. Three health states were considered: complete protection (CP), complete response at best (CR) and incomplete response (no CR). Complete protection indicates no emetic episodes, no use of rescue medication and no more than mild nausea [defined as visual analogue scale < 25 mm]. Complete response was defined as no vomiting and no use of rescue medication (with studies not defining CR in this way excluded). Incomplete response indicates experience of emesis episodes and/or rescue medications.

Patients enter the economic model on the day of chemotherapy administration. Depending on the efficacy of the administered antiemetic, patients will have different probabilities of avoiding emesis and rescue medication (CR in acute phase) or experiencing emesis and/or rescue medication (no CR in acute phase). From the second to the fifth day after chemotherapy, patients will be exposed to different probabilities of avoiding emesis and rescue medication (CR in delayed phase), as opposed to failing to achieve response (no CR in delayed phase). At the end of the cycle, the average cumulative costs and effects (quality of life) will be calculated for a given treatment arm of the model. During modelling, an episode of no CR was assumed to have a large impact on costs and quality of life. In the CP health states, it was assumed a zero cost for managing an emetic episode. The analysis was evaluated from the Spanish healthcare payers´ perspective.

The efficacy data for NEPA was derived from the registration phase III trial (NETU-07–07) [24] whose patient characteristics are reported in Table 1. The efficacy for comparators were odds ratios compared to NEPA obtained from an independent network meta-analysis performed by Abdel-Rahman et al. (Table 2) [8].

**Table 1:** Patient baseline and disease characteristics

Characteristic	PALO (*N* = 136)	NEPA_100_ (*N* = 135)	NEPA_200_ (*N* = 137)	NEPA_300_ (*N* = 135)	APR + OND (*N* = 134)
Gender (%)					
Male	57.4	57.0	57.7	57.0	56.0
Female	42.6	43.0	42.3	43.0	44.0
Median age (years)	55.0	55.0	55.0	53.0	55.5
Alcohol consumption (%)					
No	58.1	58.5	59.1	54.1	56.0
Rarely	37.1	34.8	34.3	37.8	39.6
Occasionally	4.4	6.7	6.6	8.1	4.5
Cancer type (%)					
Lung/respiratory	30.1	28.9	25.5	25.9	26.1
Head and neck	17.6	20.0	22.6	24.4	19.4
Ovarian	16.9	13.3	14.6	17.8	18.7
Other urogenital	13.2	14.1	18.2	11.1	13.4
Gastric	5.9	6.7	5.1	5.9	6.0
Other GI	7.4	3.0	5.1	4.4	7.5
Breast	2.9	8.1	4.4	5.9	5.2
Other	5.9	6.0	4.4	4.4	3.7
Karnofsky index (%)					
70%	2.9	1.5	2.9	3.0	2.2
80%	30.1	33.3	29.2	24.4	27.6
90%	58.8	57.8	54.7	60.0	61.2
100%	8.1	7.4	13.1	12.6	9.0
Chemotherapy^a^ (%)					
Cisplatin alone	15.4	15.6	14.6	14.1	14.9
Concomitant low	52.9	45.9	56.9	48.1	52.2
Concomitant moderate or high	31.6	38.5	28.5	37.8	32.8

*NEPA* netupitant and palonosetron, *APR* aprepitant, *OND* ondansetron, *GI* gastrointestinal.

^a^The median cisplatin dose was 75 mg/m^2^ for each group

**Table 2:** Response rates of NEPA and relative efficacy of the comparators

Treatment	Phase	Complete response	Complete protection
		HEC NETU-07–07 (*n* = 135)/RR (95% CI)	
NEPA	Acute	0.985 (0.965–0.999)	0.970 (0.942–0.999)
	Overall	0.986 (0.845–0.948)	0.830 (0.766–0.893)
		Comparators/OR vs NEPA (95% CI)	
APR (PO) + OND (PO)	Overall	2.23 (0.73–5.69)	1.00 (0.90–1.10)
APR (PO) + PAL (IV)	Overall	1.46 (0.84–2.46)	1.00 (0.90–1.10)
FOS (IV) + GRA (IV)	Overall	2.27 (0.66–6.15)	1.00 (0.90–1.10)

*RR* response rate, *OR* odds ratio, *CI* confidence interval, *NEPA* netupitant and palonosetron, *HEC* highly emetogenic chemotherapy, *APR* aprepitant, *OND* ondansetron, *PAL* palonosetron, *FOS* fosaprepitant, *GRA* granisetron, *PO* per os (by mouth), *IV* intravenous

The effect measures the quality of life included in the model as utilities. The utility weight is a scale from zero to one where zero is the lowest health possibly and one is perfect health. Utilities of 0.90 (95% CI 0.68–1.00) [9], 0.70 (95% CI 0.53–0.88) [9] and 0.27 (95% CI 0.18–0.30) [21] were used for CP, CR and no CR, respectively.

In the analyses, NEPA was compared with aprepitant (PO) plus ondansetron (PO), aprepitant (PO) plus palonosetron (IV), fosaprepitant (IV) plus granisetron (IV), palononostron (IV) and ondansetron (PO), all in combination with dexamethasone selected as relevant comparators on the Spanish market as well as recommended by clinical guidelines [1].

Healthcare resource utilization, i.e., proportion of patients per chemotherapy cycle including hospitalization, rescue medication, outpatient care and physician care, due to CINV, were obtained from a German-published survey including 208 patients undergoing HEC [25].

Direct costs were related to antiemetic drugs and CINV episode management, the latest being estimated from the work by Restelli et al. in an Italian setting, as no specific Spanish data were available [39]. This approach was also used in the previous adaptations for Greece and Germany [11]. Cost per hospitalization, rescue medication and physician visit were €290.31, €13.80 and €21.97, respectively. No cost for outpatient care was considered. Accordingly, the cost of CINV episode management by cycle of chemotherapy was estimated at €31.51 per patient. Drug costs were based on recommended doses from international guidelines and Spanish unit costs (ex-factory price) [2]. Generic prices, including aprepitant and fosaprepitant, were used (Table 3), and all costs were presented in 2020 euros.

**Table 3:** Cost of treatment per regimen

Treatments*	Cost per regimen (€)	Cost dex (€)	Cost IV admin (€)	Total cost (€)
NEPA (PO)	€60.00	€2.12 (IV)	NA	€62.12
APR (PO) + OND (PO)	€37.47	€2.12 (IV)	NA	€39.59
APR (PO) + PAL (IV)	€72.23	€2.12 (IV)	NA	€74.35
FOS (IV) + GRA (IV)	€64.37	€2.12 (IV)	€21.97	€88.46

General Council of Official Associations of Spanish Pharmacists [2]

*NEPA* netupitant and palonosetron, *APR* aprepitant, *OND* ondansetron, *PAL* palonosetron, *FOS* fosaprepitant, *GRA* granisetron, *dex* dexamethasone, *PO* per os (by mouth), *IV* intravenous, *NA* not applicable

*All regimens in combination with dexamethasone

Treatment-related adverse events (TRAEs) were not included in the analysis since no clinical or statistical significant differences in CINV TRAE between NEPA and the comparators were reported in the NETU trials [3, 20, 24].

The primary outcomes were incremental cost-effectiveness ratios (ICERs) for the cost per emetic event avoided and cost per quality-adjusted life days (QALDs) gained. The ICERs were calculated by dividing the difference in total costs (incremental costs) and the difference in effects (incremental emetic events or QALDs) between NEPA and the comparator treatment.

Deterministic one-way sensitivity analyses were conducted including the cost for NEPA (± 25%); cost of episode management (± 25%); utility values (95% CI); NEPA CR and CP rates in acute and overall phases (95% CI; Table 2), and odds ratios (95% CI; Table 2).

## Results

Overall treatment costs with NEPA equalled to €65.40 over the 5-day time horizon with lower total treatment costs obtained with the combination of aprepitant plus ondansetron, equalled to €46.07 (Table 4). The total costs for aprepitant plus palonosetron and fosaprepitant plus granisetron were higher with €78.92 and €95.03, respectively, primarily since these treatment combinations are requiring additional IV administration costs. For CINV, episode management cost and cost for the acute and delayed phases, NEPA accrued the lowest costs among the comparators. The differences in accumulated QALDs were low between the treatments ranging from 4.117 days for aprepitant plus ondansetron to 4.272 days for NEPA. Patients in the NEPA arm were predicted to have more emesis-free and CINV-free days than the comparators (Table 3).

**Table 4:** Base case analysis of NEPA compared to other recommended treatments

	**NEPA (PO)**	**APR (PO) + OND (PO)**	**APR (PO) + PAL (IV)**	**FOS (IV) + GRA (IV)**
Costs (€)				
Treatment drug	62.12	39.59	74.35	88.46
CINV episode management	3.28	6.48	4.57	6.57
Inpatient care	3.02	5.97	4.21	6.05
Rescue medication	0.14	0.28	0.20	0.29
Physician care	0.11	0.23	0.16	0.23
Cost in acute phase	0.47	1.03	0.69	1.05
Cost in delayed phase	2.80	5.44	3.88	5.52
Total costs	65.40	46.07	78.92	95.03
Health outcomes				
Average emesis-free days	4.703	4.393	4.580	4.384
Average CINV-free days	4.500	4.393	4.500	4.384
Emesis-free patients (%)	89.6%	79.4%	85.5%	79.1%
Emetic events (estimate)	0.60	1.19	0.84	1.20
CINV-free patients (%)	83.0%	79.4%	83.0%	79.1%
Quality-adjusted life days	4.272	4.117	4.220	4.112
Quality-adjusted life years	0.0117	0.0113	0.0116	0.0113
Cost/outcomes				
Cost per avoided emetic event	-	€33	Dominant	Dominant
Cost per QALD gained		€125	Dominant	Dominant

*CINV* chemotherapy-induced nausea and vomiting, *NEPA* netupitant and palonosetron, *APR* aprepitant, *OND* ondansetron, *PAL* palonosetron, *FOS* fosaprepitant, *GRA* granisetron, *PO* per os (by mouth), *IV* intravenous

In summary, the results of the analysis showed that NEPA was dominant against aprepitant combined with palonosetron and fosaprepitant combined with granisetron (Table 4), while compared to generic aprepitant plus ondansetron, NEPA leads to an incremental cost per avoided emetic event of €33 and cost per QALD gained of €125.

**Fig. 2:**
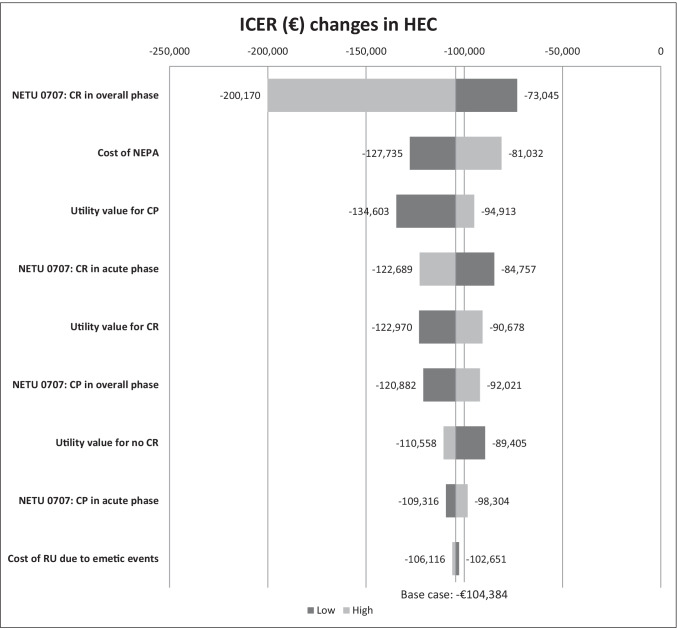
Tornado diagram including results from deterministic one-way sensitivity analyses. ICER, incremental cost-effectiveness ratio; NETU 0707, efficacy data from trial; CR, complete response; CP, complete protection; RU, health resource utilisation; HEC, highly emetogenic chemotherapy

In the one-way sensitivity analysis, the CR rate for NEPA in the overall phase was found to be the most influential parameter followed by the cost of NEPA (Fig. 2).

## Discussion

The results of the base case analysis showed that NEPA was *dominant* against aprepitant plus palonosetron and fosaprepitant plus granisetron and *cost-effective* against aprepitant plus ondansetron, with an ICER per cost per avoided emetic event of €33 and cost per QALDs gained of €125. Price of generic aprepitant and fosaprepitant was used in the analysis. The small QALD gained estimates in the analyses may be explained by the transient nature of CINV, and the fact that treatments were not assumed to have an impact on survival.

Beside the input of aprepitant and fosaprepitant generic prices, the results presented for Spain were in line with the results previously presented for Germany, Greece [11] and Singapore [31]. In Germany, NEPA was dominant against all comparators (i.e., aprepitant plus ondansetron, aprepitant plus palonosetron, fosaprepitant plus granisetron and rolapitant plus granisetron), being the cheapest with a total cost of €81.49 and the most effective with total QALDs of 4.272 [11]. NEPA was also dominant against all comparators in Greece (aprepitant plus ondansetron, aprepitant plus palonosetron and fosaprepitant plus granisetron), with a total cost of €85.00. Therefore, it was concluded that NEPA was a cost-effective strategy for prevention of CINV in patients undergoing HEC in both Germany and Greece. In Singapore, the results of the analysis showed that NEPA was dominant against aprepitant plus ondansetron, aprepitant plus palonosetron and fosaprepitant plus granisetron and palonosetron, and cost-effective against ondansetron, with an ICER of 47 SGD (€35) per avoided emetic event and 53,244 SGD (€40,073) per QALY gained [31].

Comparing the results from different studies poses various challenges, as depicted in CINV cost studies performed in Europe [32, 43] and the USA [12]. A study assessed the direct costs of CINV in three European countries by means of a survey covering the management and resource utilization, from patients experiencing a CINV episode during the 6-month period preceding the survey [43]. The mean cost per patient per severe CINV episode resulted in approximately €389 in Italy, €750 in France and €1017 in Germany [43]. A study from the USA reported a mean (standard deviation) cost of CINV visits of $5299 ($6639); for inpatient, $7448 ($7271); outpatient, $1494 ($2172); and emergency room, $918 ($1071) and the mean per-patient CINV-associated costs across all patients were $731 ($3069) [12]. The higher cost, presented in this study, can be explained by the fact that costs were included from the chemotherapy administration date to 30 days later, while our study was restricted to the first 5 days. Similar to the current study, Guiliani et al. [19] concluded that NEPA plus dexamethasone was cost-effective in HEC and MEC in a trial-based study. Botteman et al. [10] conducted a cost-effectiveness study using patient-level data from a phase III trial and concluded that NEPA was highly cost-effective versus an aprepitant-based regimen in post-HEC prevention. Finally, consistent results were shown also in the US setting where Park et al. showed that beside increase in acquisition cost, the introduction of NEPA into a US formulary would lead to a net decrease in the total budget due to substantial reduction in CINV event-related resource utilzation and medical cost savings for the healthcare payers [36].

The study presents several limitations. Age and sex are known risk factors for occurrence of CINV [37]. However, it was assumed that the differences in age and sex had no impact on efficacy and cost given that the target population was the general population receiving HEC for cancer treatments. Further, no Spanish data for the healthcare resource utilisation was available that may suggest an underestimation of the clinical and economic burden of CINV. The meta-analysis used for estimating comparator’s efficacy contained only odds ratios in the overall phase setting and for CR; hence, assumptions of similar differences between the acute and overall phase, as well as equal efficacy between comparators and NEPA in CP, had to be made. Finally, broad credible intervals were presented for the odds ratios in the meta-analysis possibly due to the presence of some degree of heterogeniety and selection bias of studies [8], and therefore, the mean values with 95% confidence intervals were used instead.

## Conclusion

In agreement with previously published cost-effectiveness and budget impact analyses in several countries, this economic evaluation demonstrates that NEPA is a dominant or cost-effective treatment alternative by most calculations to current antiemetic standards of care in Spain during 5 days of chemotherapy treatment in cancer patients.

## Data Availability

Not applicable.
